# Increased HA/CD44/TGFβ signaling implicates in renal fibrosis of a Col4a5 mutant Alport mice

**DOI:** 10.1186/s10020-025-01146-0

**Published:** 2025-03-12

**Authors:** Yantao Bao, Weiqing Wu, Jiyun Lin, Yuankai Yang, Sheng Lin, Jindi Su, Yueyuan Qin, Baojiang Wang, Shan Duan

**Affiliations:** 1 Shenzhen Maternity and Child Healthcare Hospital, Southern Medical University, Shenzhen, 518000 Guangdong China; 2 Shenzhen Key Laboratory of Maternal and Child Health and Diseases, Shenzhen, 518000 Guangdong China; 3 Shenzhen Health Development Research and Data Management Center, Shenzhen, 518028 Guangdong China; 4 Laboratory of Molecular Medicine, Institute of Maternal and Child Medicine, Shenzhen Maternity & Child Healthcare Hospital, Shenzhen, 518040 Guandong China

**Keywords:** XLAS, COL4A5, Renal fibrosis, Hyaluronan, CD44, HAS2

## Abstract

X-linked Alport syndrome (XLAS) caused by X-linked COL4A5 gene mutation is a hereditary disease that affects mainly the kidney. XLAS patients, especially males whose single copy of the COL4A5 gene is disrupted, suffer from a life-threatening renal disease, the mechanism of which remains unclear. Renal fibrosis is a characteristic pathology observed in XLAS kidney tissue. However, the molecular path from COL4A5 loss-of-function to fibrotic pathology is largely unknown. On the basis of a previously established XLAS mouse model, our study revealed an activated CD44-TGFβ signaling known to strongly promote fibrosis, along with an increased level of low molecular weight hyaluronan (LMW-HA) instead of high molecular weight hyaluronan (HMW-HA), to activate CD44-dependent TGFβ signaling in XLAS renal tissues. Additionally, hyaluronan synthase 2 (HAS2), an enzyme primarily responsible for HA production, was found to be upregulated in XLAS. In particular, in vitro studies revealed that COL4A5 knockdown in human kidney-derived HEK-293 cells can upregulate HAS2 at both the RNA and protein levels. The novel contribution of our study is finding that COL4A5 deficiency may lead to HAS2 overexpression and HA accumulation to activate CD44-TGFβ signaling, thereby promoting fibrosis, possibly suggesting that HAS2 and CD44 are potential therapeutic targets for impeding renal fibrosis in XLAS.

**Supplementary Information:**

The online version contains supplementary material available at 10.1186/s10020-025-01146-0.

## Introduction

Renal damage is the most life-threatening symptom of X-linked Alport syndrome (XLAS), an inherited disease caused by X-linked COL4A5 gene mutations, which cause more than 80% of all Alport syndrome cases (the remaining cases are caused by COL4A3/COL4A4 gene mutations) (Kruegel et al. 2013). Therefore, preventing renal disease is a high priority of XLAS treatment in the clinic. Owing to X-linked heredity, hemizygous XLAS males tend to develop more severe renal symptoms and higher risks (90% males versus 16% females by age 40 years) of developing end-stage renal disease (ESRD) than do heterozygous females (Kruegel et al. 2013; Yamamura et al. 2017; Kashtan 2019). Unfortunately, treatment options for XLAS-related renal diseases are still limited. Knowledge of the pathways underlying the pathological development of XLAS-related renal disease is the key to uncovering novel therapeutic targets, and therefore, this issue requires further exploration.

Fibrosis is one of the main pathological characteristics of AS-related kidney diseases (Yamamura et al. 2017; Warady et al. 2020) and occurs at both the glomerulus and tubulointerstitial (Fogo and Raymond, 2025), resulting in extracellular matrix hypertrophy, causing extensive damage to renal functions. Hyaluronic acid (HA) is a critical component of the extracellular matrix (ECM) and is strongly associated with the progression of fibrosis in multiple organs, including the lung, liver and kidney (Xia et al. 2021; Yang et al. 2019; Kaul et al. 2022). The HA molecule is composed of linearly repeating (glucuronate-β 1,3-N-acetylglucosamine-β 1,4-) residues, and thus, its molecular weight varies ( Rabelink et al. 2024). The biological effects of HA differ depending on its molecular weight (Kaul et al. 2022). Generally, low-molecular-weight (LMW, generally < 300 kDa) HA, rather than high-molecular-weight (HMW, > 1000 kDa) HA, can strongly promote fibrosis (Kaul et al. 2022; Yang et al. 2019; Liu et al. 2019). By producing HA, hyaluronan synthase (HAS), especially HAS2, has also been found to increase HA production and thereby drive fibrosis (Yang et al. 2019; Selbi et al. 2006). However, the role of HAS2 in Alport kidney disease has rarely been investigated.

CD44, which acts as a canonical HA receptor, is strongly associated with fibrosis (Acharya et al. 2008; Yang et al. 2019; Midgley et al. 2017; Xia et al. 2021). Interestingly, increasing evidence has revealed increased levels of CD44 in the kidney upon injury (Sun et al. 2024), nephritis (Eymael et al. 2018) and other pathological conditions, which are associated with the pathology of renal fibrosis. All of the above evidence suggests that a biological process from HA production to an increased level of HA and then activation of its receptor CD44 might be associated with fibrosis. Nonetheless, the role of HA/CD44 in XLAS-related renal fibrosis is unexplored.

TGFβ signaling is one of the best-known pathways that promotes mesenchymal transition and fibrosis (Meng, Nikolic-Paterson, and Lan 2016). TGFβ comprises three isoform transcripts that are implicated in fibrosis (Meng, Nikolic-Paterson, and Lan 2016; Sun et al. 2021). In addition, activation of TGFβ signaling has long been observed in Alport syndrome patients (Gomes et al. 2022). A ramipril therapy that mainly targets the renin‒angiotensin‒aldosterone system (RAAS) to ameliorate AS-related kidney arterial hypertension effectively reduces TGFβ levels and thereby inhibits renal fibrosis (Gross et al. 2003), suggesting an important role of TGFβ in renal fibrosis.

Pathological COL4A5 mutations generally lead to either a loss of expression or a loss of function of its encoded α5 collagen chain, an indispensable component of the α3α4α5 trimer, which forms the glomerular basement membrane (GBM) (Kruegel et al. 2013). This change directly compromises glomerular function and initiates XLAS pathology. However, the mechanistic routes from Col4a5 deficiency to fibrotic pathology remain unknown.

### Novel contribution of this study

On the basis of a previous Col4a5-deficient mouse model that mimics clinical XLAS renal symptoms (Wu et al. 2023), we discovered notable upregulation of CD44 and TGFβ, together with substantial accumulation of HA in the renal tissues of Col4a5 mutant mice compared with those of healthy controls. In addition, HA was found to activate CD44 and then TGFβ, which is a dynamic driver of fibrosis. Moreover, HAS2 was upregulated in Col4a5 mutant mouse kidney tissues, and knockdown of COL4A5 expression in human kidney-derived cells may directly upregulate HAS2. Our study suggested that an HA/CD44/TGFβ axis triggered by HAS2 overproduction due to COL4A5 deficiency could be strongly implicated in XLAS renal fibrosis.

## Materials and methods

### Antibodies and reagents

Antibodies and chemicals used in this study were purchased as follows: CD44 rabbit polyclonal antibody (Proteintech, #15675, 1:1000 dilution for WB and 1:100 for IF); TGFβ1 + 2 + 3 rabbit polyclonal antibody (Solarbio, #K009638P, 1:500 for WB); HAS2 and GAPDH mouse monoclonal antibody (Santa Cruz, sc-365263, 1:500 dilution for WB and 1:100 for IF, sc-365062, 1:1000 for WB); COL4A5 rabbit polyclonal antibody (GENETEX, #GTX56030, 1:1000 for WB); DyLight 800 anti-mouse secondary and DyLight 680 anti-rabbit secondary antibodies (Immunoreagents, #GTXMu-003-D800NHSX, #GTXRb-003-D680NHSX, 1:10000 for WB); LMW Hyaluronic acid (Sangon biotech, #A003907); HMW Hyaluronic acid (Sigma, #73641); Biotinylated Hyaluronic Acid Binding Protein (Millipore, # 385911, 1:100 for IF), Alexa Fluor™ 647 Conjugates of Streptavidin (Thermo, S21374, 1:500 for IF); Cy3 conjugated Sambucus Nigra Lectin (SNL Cy3, Vector Laboratory, for GBM detection, CL-1303); HRP conjugated Streptavidin, Alexa Fluor™ 488/594 conjugated anti-mouse and anti-rabbit secondary antibody (Servicebio, #G3431, #GB28303, #GB25303, 1:500 for IF).

### Animal samples and cell culture

This study employs a previously produced Col4a5 mutant C57BL/6 mice harboring a NM_000495.5(COL4A5) c.980_983delATGG deletion that was recently identified in a Chinese XLAS family (Wu et al. 2023). Hemizygous male mice were described to develop ESRD with apparent edema by age of 28 weeks with significant pathological signs and molecular alterations and die before age of 32 weeks, contrasting the female carriers with milder symptoms and their biochemical parameter less affected (Wu et al. 2023). These mice model highly mimic the renal symptoms of XLAS and thus were investigated in this study. We have only access to a total 9 pairs of kidney tissues from 28-week-old hemizygous mutant and healthy control male mice, with identical age and gender to those for RNAseq test. These tissues were previously stored in liquid nitrogen or paraffin-embedded after fixated by 4% Paraformaldehyde (PFA) solution upon fresh dissection. Randomization and blinding were employed in this study by concealing sample information from the experiment conductor. All animal experiments were approved by the Animal Ethics Committees of Shenzhen Maternity and Child Healthcare Hospital.

A human embryotic kidney-derived epithelial-like cell line HEK-293 and a human mesenchyme-derived fibroblast cell line HEPM (obtained from ATCC) were used in this study as representatives of kidney parenchymal and mesenchymal cells, respectively. Both cells were cultured using Dulbecco’s Modified Eagle Medium supplemented with 10% FBS, under a humidified condition of 37℃, 5% CO2 in a Thermo incubator. Culture medium was refreshed every 24 h, and cell passages were performed once the cell coverage reached approximate 90%.

### RNAi and transfection

19nt siRNA sequences (listed in supplementary materials) targeting human gene transcripts CD44 and COL4A5 were obtained by using a DSIR online designing tool (Vert et al. 2006), and were synthesized by Sangon Biotech (Shanghai) Co., Ltd. siRNA transfection was conducted using a Lipofectamine RNAiMAX kit (Invitrogen) following the manufacturer protocol. Cells were collected 72 h after siRNA transfection for further experiments. A CD44 ORF expressing plasmid were purchased from Sino Biologicals, which were amplified in a DH5α E.Coli (Tiangen) and harvested using an Endo Free Maxi Kit (Tiangen). Plasmid was transfected using a PEI-4 K reagent into target cells which were cultured overnight to reach more than 80% of coverage. Cells were collected for further detection 48 h post plasmid transfection.

### RNA isolation and realtime quantitative PCR

RNA was extracted from tissues and cells by using a Nucleispin RNA Plus isolation kit (MN). RNA concentrations were determined using Nanodrop (Thermo). 1 μg RNA was used to synthesize the first strand cDNA by a AG1161 Evo M-MLV Plus 1st Strand cDNA Synthesis Kit (AG biotech). cDNA samples were tested by real-time quantitative PCR in a LightCycler 96 instrument (ROCHE) using a SYBR Green Pro Taq HS premix (AG biotech). Procedures were carried out following the manufacturer’s instruction. PCR Primers were listed in supplementary materials.

### Protein sample preparation

Kidney samples freshly stored in liquid nitrogen were quickly retrieved on ice. After measuring the tissue weight, immediately added 1 ml RIPA buffer and homogenized the tissue using a Qiagen tissueruptor. Samples were then subject to ultrasonic vibration for 2 min and then centrifuged by 13,000 g for 15 min. Supernatants (protein samples) were transferred into clean tubes. Protein concentrations were determined using a Bradford method (Thermo) and were standardized for detection. Samples were then mixed with loading buffer (Beyotime) and further boiled on a 99℃ incubator for 10 min.

For protein samples extracted from cells, Calculated 10^6^ cells were collected and centrifuged (1000 g, 5 min) in pellets. Wash the pellets by PBS twice and resuspend with a 2× SDS loading buffer (Beyotime) with a Halt Protease Inhibitor Cocktail (Pierce). Samples were thoroughly homogenized by pipetting repeatedly, heated on a 95℃ incubator for 30 min and centrifuge at 13,000 g for 10 min to discard insoluble pellets.

### Western blot

Above protein samples were loaded on an 8% SDS-PAGE gel for electrophoresis using a tris-glycine buffer (25 mM Tris, 0.2 M Glycine) containing 1% SDS. After the electrophoresis, proteins were transferred to a 0.45 μm nitrocellulose membrane in the same tris-glycine buffer supplemented with 10% methanol using a Bio-rad Trans-Blot® suite submerged in ice. After transferring, membranes were incubated in a 5% BSA blocking buffer at room temperature (RT) for 1 h on a horizonal shaker. Primary antibodies were administrated for incubation at 4℃ on a gentle shaker overnight. The next day membrane was washed by a TBS-T buffer for three times (10 min each time), followed by another incubation using host specific secondary antibodies at RT for 1 h. After incubation, the membranes were washed again by TBS-T buffer for three times. Protein bands were visualized in an Odyssey Imaging Scanner (Li-COR) and further analyzed by an Image-J software.

### HA content determination in mouse kidney

The HA in kidney tissue lysates were isolated and measured. The method of separating different molecular sizes of HA from tissue lysates were described before (Yang et al. 2019). Concisely, tissue lysates were pre-treated with a Pronase (Millipore) on a 60℃ incubator for 4 h to breakdown unnecessary proteins and then heated at 100℃ for 30 min to deactivate Pronase. Samples were loaded on an ultrafiltration tube (Satorius) with a molecular size screen of 300 kD, yielding two fractions of samples containing HA < 300 kD and HA > 300 kD. HA concentration in each diluted samples were determined by an ELISA method (Solarbio). The final HA content was standardized based on the corresponding tissue weight. Whole HA content was calculated by summing up both < 300 kD and > 300kD HA fractions.

### Immunostaining

Tissue slides were prepared as previously described (Wu et al. 2023). Dewaxed and rehydrated slides were bathed in a citrate antigen retrieval solution (Servicebio) and heated in microwave for 20 min. Slides were washed by PBS three times, treated by 3% H_2_O_2_ to block peroxidase, and again washed by PBS three times. 5% BSA in PBS were administrated to cover the whole tissue of each slide, and incubate at RT for 30 min for blockage. For immunohistochemistry, a HABP protein was used as probe of HA (Šínová et al. 2021). Biotinylated-HABP diluted in 3% BSA were applied to the tissues, incubate at 4℃ in a moist atmosphere overnight. The next day, Slides were washed by PBS, subject to a HRP Conjugated Streptavidin (Servicebio), incubated at RT for 1 h and re-washed. A DAB solution was applied to the sample slides and allowed to react for 30 s before immediately eliminated by washing the slides. Slides were then re-stained by Hematoxylin, washed, oven dried and sealed by coverslips. For fluorescence staining of multiple targets, a tyramide signal amplification (TSA) triple staining kit (Servicebio) were used. Briefly, after first-round of staining, Tyramide-H_2_O_2_ mixture was administrated to cover the tissue. Slides were incubated in dark at RT for 10 min, washed by PBS, underwent another antigen retrieval and PBS wash. Same procedure is employed for third-round staining. GBM was stained using a SNL which binds to disaccharide, a rich component in GBM(Song et al. 2020). Slides were then restained by DAPI (Servicebio), sealed by coverslips, scanned in a Pannoramic MIDI (3DHISTECH) and imaged using a CaseViewer2.4 software.

### Statistical analysis

Tissue sample testing was technically triplicated, while cell experiments were independently carried out three times. Data was presented as mean ± SD and tested for normality before performing the statistics. A two-way ANOVA method followed by a Tukey’s multiple comparison test was conducted by a SPSS software for statistical significance determination between each group. Significances were indicated as follows: *, *P* < 0.05, **, *P* < 0.01, ***, *P* < 0.001, ns, no significance.

## Results

### CD44 and TGFβ are markedly upregulated in XLAS mouse kidneys

**Fig. 1:**
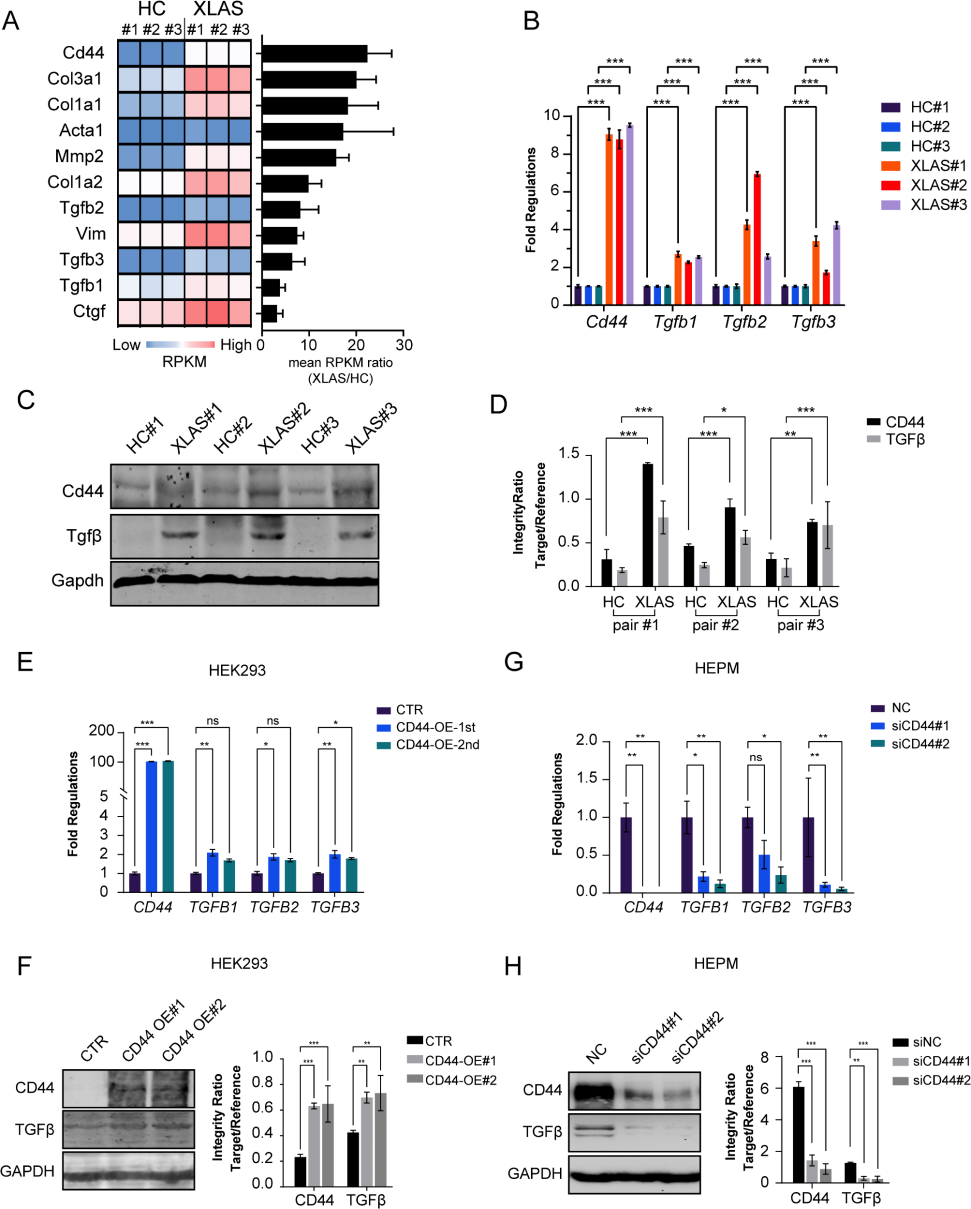
CD44 and TGFβ are remarkably up-regulated in XLAS mice kidney. (**A**) Increased expression levels of selected fibrotic marker genes based on RNA-sequencing data of XLAS vs. healthy control (HC) mice kidney, data presented as mean ± SD. Expressions of Cd44, Tgfb1, Tgfb2, Tgfb3 gene transcript were validated by qPCR, data presented as mean ± SD, *n* = 3 wells, (**B**) in RNA extracts, and protein levels of Cd44, Tgfb1, Tgfb2, Tgfb3 detected by WB (**C**) and quantified as column graph (**D**) in protein extracts from freshly stored tissues, data presented as mean ± SD, *n* = 3 experiments. Increased RNA (**E**) and protein (**F**) levels of Tgfb upon CD44 overexpression, and decreased RNA (**G**) and protein (**H**) levels of Tgfb by CD44 RNAi, data presented as mean ± SD, *n* = 3 experiments

Compared with those of healthy controls, XLAS mouse kidneys presented with widespread fibrosis (Fig. S1). To investigate the mechanism underlying renal fibrosis, we used RNA-seq data described previously (Wu et al. 2023) to screen differentially regulated genes involved in fibrosis in kidney tissues between hemizygous Col4a5-mutant male mice (XLAS) and healthy control males (HC). As expected, the expression of fibrotic markers, such as Col1a1, Col1a2, Col3a1, and Acta1, was upregulated in the XLAS model mice compared with the healthy controls (Fig. 1A). Notably, Cd44 was the most strongly upregulated gene of these markers (Fig. 1A). In addition, three Tgfβ transcripts, namely, *Tgfb1*,* Tgfb2*, and *Tgfb3*, were also upregulated (Fig. 1A). The expression levels of CD44 and Tgfβ in Col4a5 mouse kidneys were further validated by qPCR and WB (Fig. 1B-D).

A previous study showed that CD44 could activate TGFβ. Therefore, we overexpressed CD44 in the human embryotic kidney cell line HEK-293 (with low CD44 expression levels, Fig. S2) and found that CD44 activated TGFβ expression (Fig. 1E, F). CD44 knockdown in a mesenchymal fibroblast line, HEPM (with high CD44 expression levels, Fig. S2), significantly silenced the expression of TGFβ (Fig. 1G, H). These results suggest that CD44 is a key factor involved in renal fibrosis in Col4a5 mutant XLAS model mice and that increased CD44 can activate TGFβ, which is a robust fibrosis promoter.

### Elevated HA levels are observed in XLAS model mouse kidney tissues

**Fig. 2:**
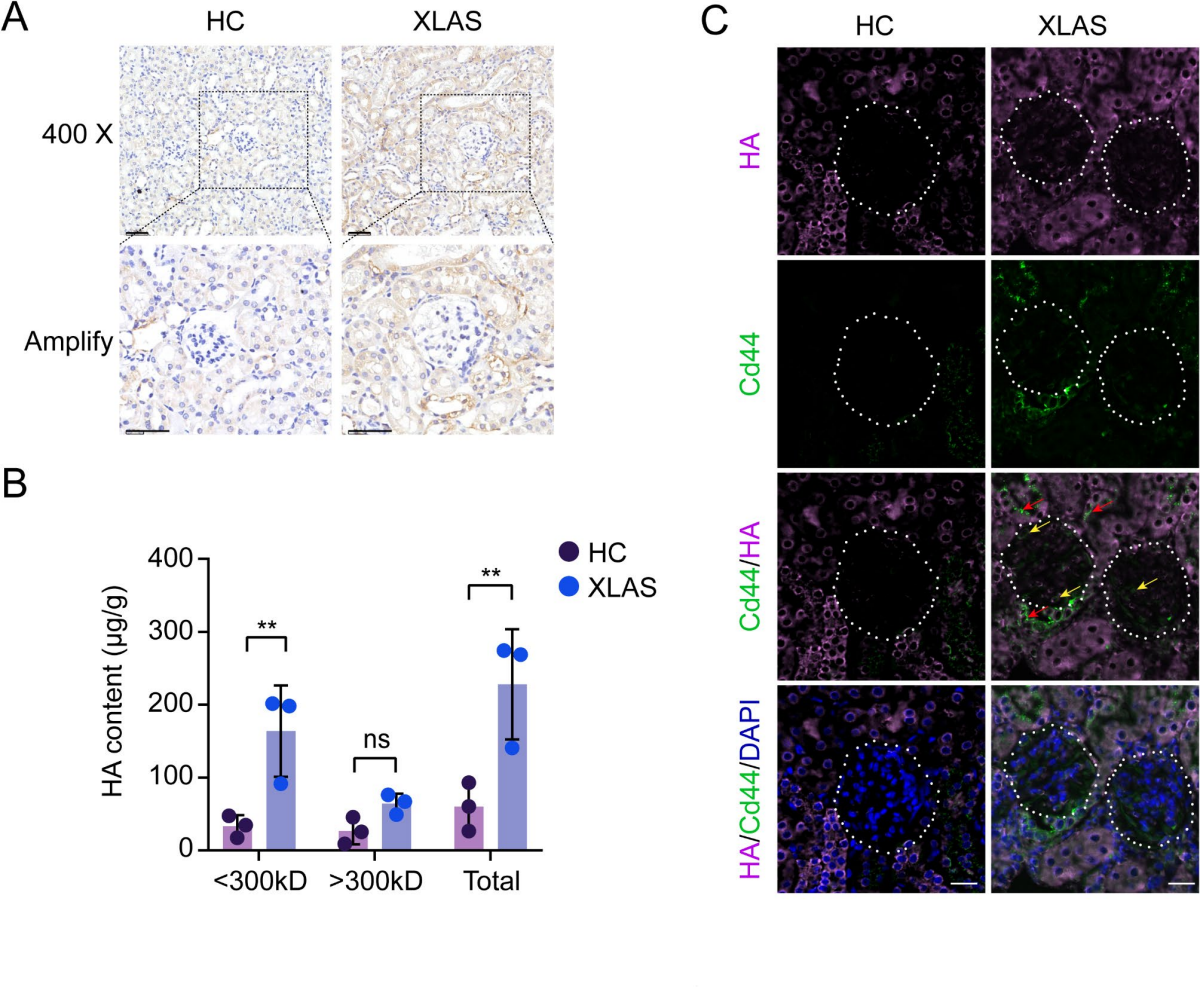
Elevated HA levels are observed in XLAS mice kidney tissues. (**A**) IHC staining of HA using a biotylated HA binding protein in XLAS vs. HC mice kidney tissues, scale bar = 50 μm. (**B**) ELISA quantification of LMW HA (< 300 kD) and HMW (> 300kD) fractioned from kidney tissue lysates, data presented as mean ± SD, *n* = 3 individual mice. (**C**) Immunofluorescence of HA (staining pink) and its receptor CD44 (staining green) in XLAS vs. HC kidney tissues, red and yellow arrows indicate increased CD44 signals in interstitial and glomerular areas of XLAS tissues, scale bar = 25 μm

Renal HA levels were subsequently investigated. We observed a substantial increase in HA levels in both glomerular and interstitial tissues in the mice with XLAS compared with the healthy controls (Fig. 2A). In addition, LMW HAs < 300 kDa and HAs > 300 kDa from kidney tissue samples were subfractured by ultrafiltration and then separately tested by ELISAs. Compared with those in the healthy controls, significant increases in total and LMW HA (< 300 kDa) levels were detected in the mice with XLAS (Fig. 2B). However, HA levels > 300 kDa, although increased, were not significantly different from those of the healthy controls (Fig. 2B). Moreover, immunofluorescence staining of renal CD44 and HA revealed increased expression of CD44 and HA in the renal tissue of the mice with XLAS compared with the control renal tissues (Fig. 2C). In addition, the signals of CD44 and HA were partially colocalized within both the glomerulus and interstitial tissues of the mice with XLAS (Fig. 2C). Notebly, Fig. 2A and C consistently demonstrated that HA and CD44 were dominantly increased in tubulointerstitial tissues instead of glomerulus, although remarkable increases of HA/CD44 signals in glomerulus were also observed. These results suggested that total HA, especially LMW HA, is markedly increased in XLAS model mouse kidney tissues and that the accumulated HA is accessible by its receptor CD44 for interaction/activation.

### Low-molecular-weight HA activates TGFβ through CD44

**Fig. 3:**
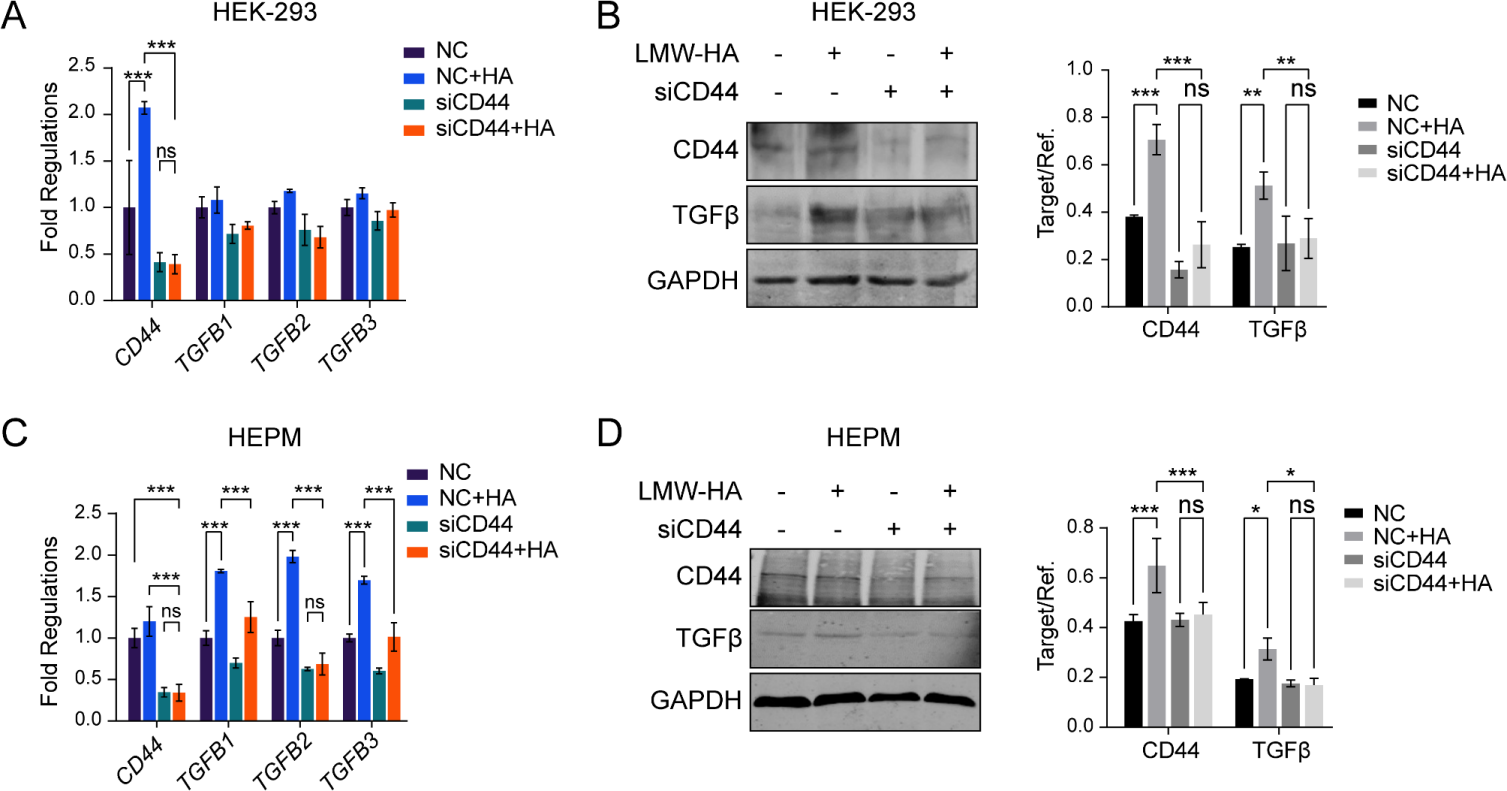
Low molecular weight HA activates TGFβ through CD44. (**A**) RNA and (**B**) protein levels of CD44, TGFB1, TGFB2 and TGFB3 in CD44 RNA interfering and negative control HEK-293 cells with and without LMW-HA treatment in culture medium, (**C**) RNA and (**D**) protein levels of CD44, TGFB1, TGFB2 and TGFB3 in CD44 RNA interfering and negative control HEPM mesenchymal cells with and without LMW-HA treatment in culture medium, data presented as mean ± SD, *n* = 3 experiments

To determine whether HA increased the expression levels of CD44 and TGFβ, exogenous LMW or HMW HA was administered to the culture media of HEK-293 and HEPM cells. We observed that LMW HA increased the protein levels of CD44 and TGFβ in both HEK-293 (Fig. 3A, B) and HEPM cells (Fig. 3C, D). However, HMW HA did not change either CD44 or TGFβ expression levels (Fig. S3). The increase in CD44 induced by LMW HA was statistically significant in both cell lines. In comparison, increases in the mRNA levels of TGFβ were significant in HEPM cells but not in HEK-293 cells, although increases in the TGFβ protein levels were significant in both cell lines. Additionally, the increases in CD44 and TGFβ in both HEK-293 and HEPM cells were abolished by CD44 knockdown (Fig. 3A-D). These results suggested that LMW HA increased CD44 expression and that TGFβ activation by LMW HA is dependent on CD44. These results also suggested that LMW HA may activate CD44/TGFβ more effectively in mesenchymal fibroblasts which express high levels of CD44.

### Col4a5 deficiency increases HAS2 expression

**Fig. 4:**
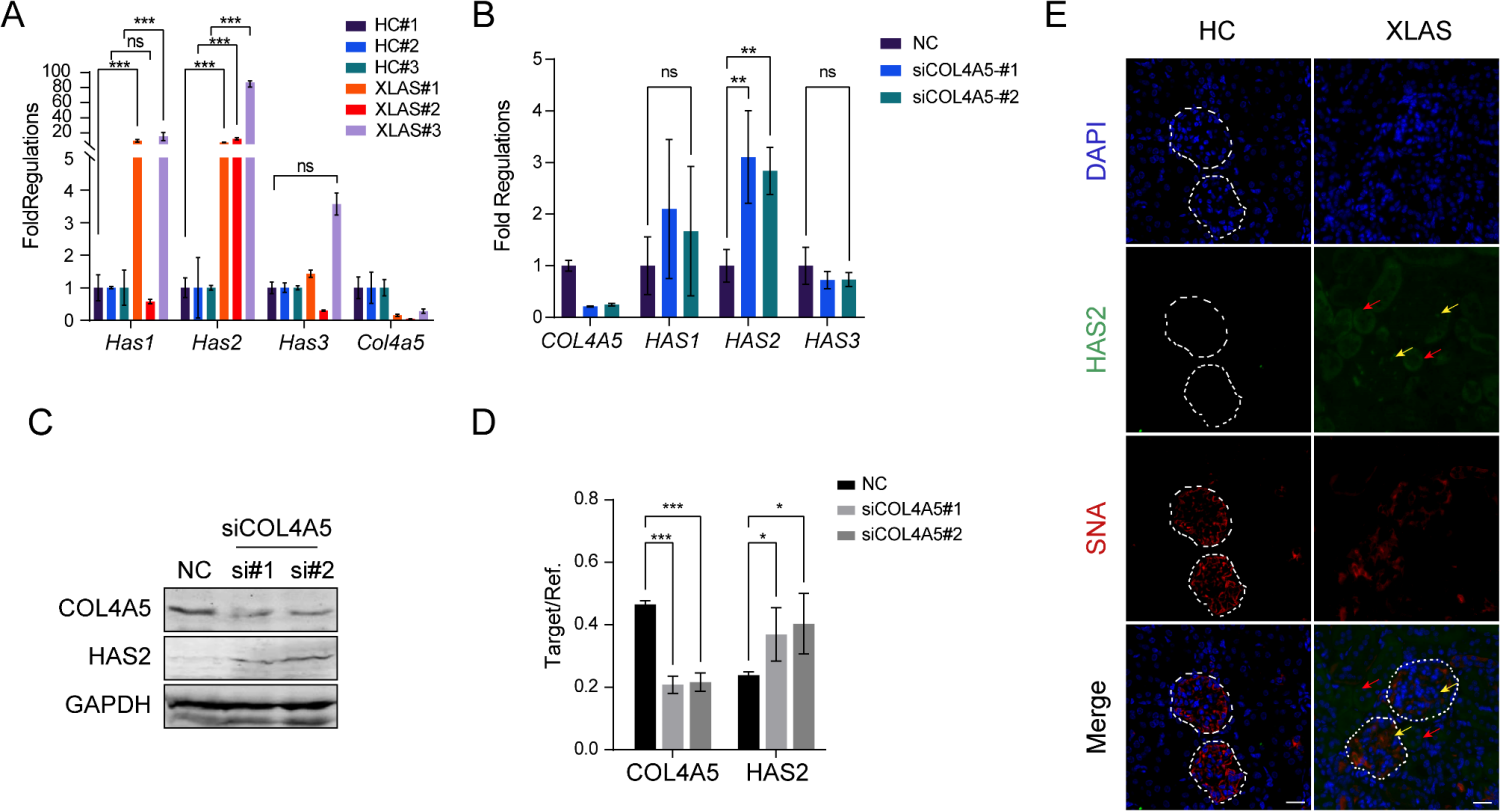
Col4a5 deficiency increases HAS2 expression. (**A**) RNA-sequencing data showed increased expressions of Has1 and Has2 in XLAS kidney; data presented as mean ± SD, *n* = 3 wells. (**B**) COL4A5 knockdown led to significant HAS2 overexpression at RNA levels (qPCR) in HEK-293 cells, data presented as mean ± SD, *n* = 3 experiments. Blot images (**C**) and band signal quantification (**D**) showed significantly increased HAS2 protein levels by COL4A5 knockdown, data presented as mean ± SD, *n* = 3 experiments. (**E**) Immunofluorescence signal of HAS2 (green) overexpression resides in both glomerulus (yellow arrow) and interstitial epithelial cells (red arrow), Sambucus Nigra Lectin (SNA) indicating GBM was stained in red and DAPI in blue, scale bar = 25 μm

**Fig. 5:**
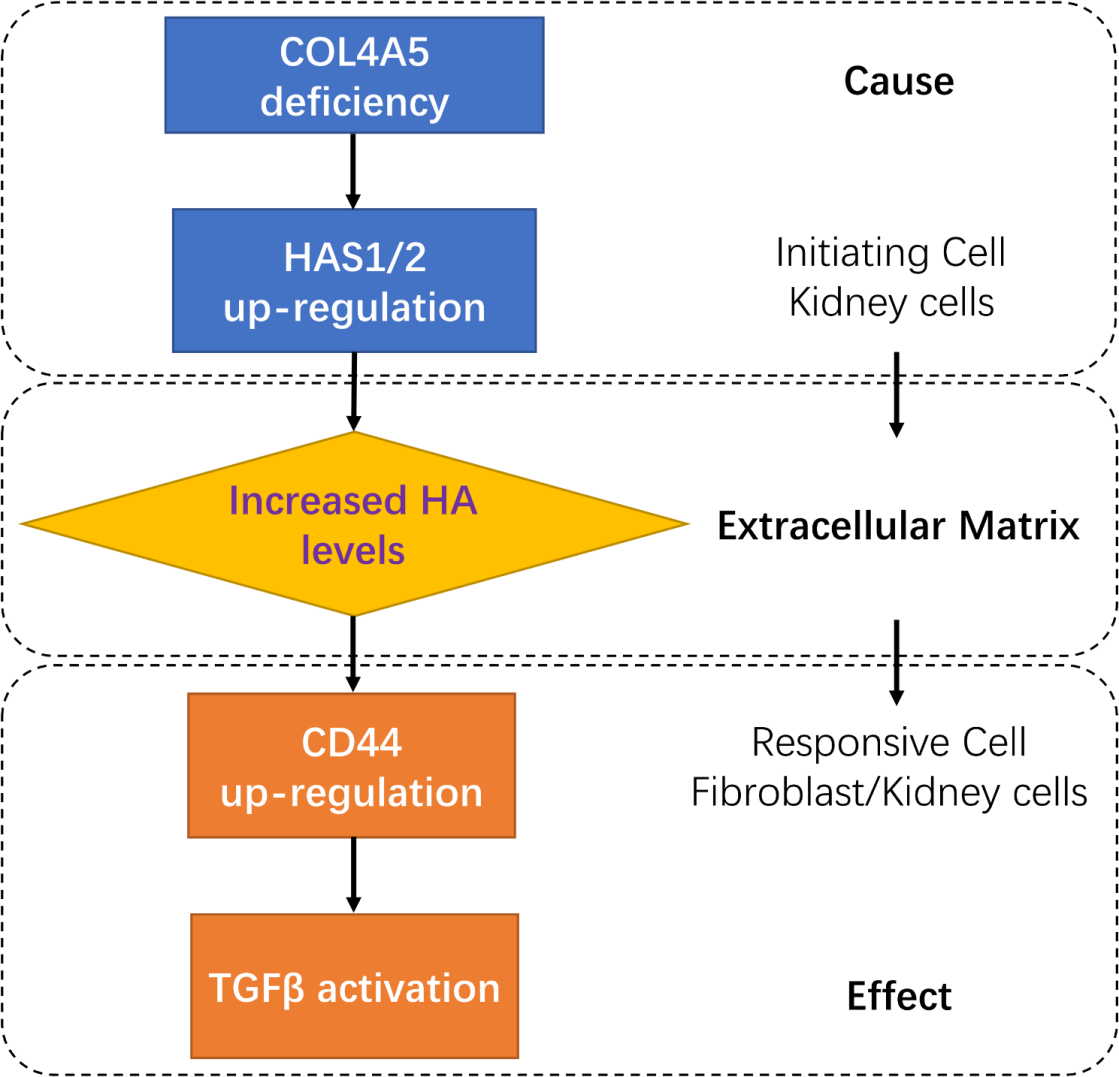
Pathological model based on XLAS mice demonstrating HAS2 up-regulation due to Col4a5 loss and HA accumulation may activate CD44/TGFβ signaling which promotes fibrosis

We then investigated the expression levels of HAS, which produces HA. Higher expression levels of HAS1 and HAS2, but not HAS3, were detected in Col4a5 mutant XLAS model mice via RNA-sequencing (Fig. 4A), suggesting a potential relationship between Col4a5 mutation and HAS overexpression. Therefore, we knocked down COL4A5 by siRNA interference in HEK-293 cells, which express the COL4A5 gene. HAS2 mRNA expression was significantly greater in the COL4A5-silenced cells than in the control cells (Fig. 4B). In comparison, the mRNA level of HAS1 increased but was not significantly different after COL4A5 knockdown, while HAS3 expression was barely changed (Fig. 4B). At the protein level, COL4A5 knockdown led to a significant increase in HAS2 (Fig. 4C, D). In addition, an immunostaining test confirmed the substantial increase in HAS2 expression in both the glomerulus and interstitial tubules in XLAS mouse kidney tissues compared with healthy tissues, where HAS2 was barely detected (Fig. 4E). These results suggested that COL4A5 deficiency may lead to the overexpression of HAS2, which produces HA, which might contribute to the early accumulation of HA and thereby activate HA/CD44/TGFβ signaling to promote fibrosis (Fig. 5).

## Discussion

Loss of the functional α3α4α5 collagen trimer causes decomposition of the GBM, leading to loss of podocyte and glomerular function, glomerulosclerosis, a thickened matrix, renal hypertension, and ultimately ESRD. In addition, our study revealed that COL4A5 deficiency may result in the upregulation of HAS2, suggesting another mechanism in which increased HAS2 expression due to Col4a5 loss may cause the overproduction of HA and thereby increased HA/CD44/TGFβ signaling. This alteration probably occurs at the very beginning when an impact of COL4A5 deficiency occurs, initiating XLAS renal pathogenesis.

Increased expression of HAS2 and HA has been reported in ischemia-induced renal fibrosis (Selbi et al. 2006). Similarly, our study revealed that HAS2 is upregulated in XLAS renal tissues, suggesting that increased HAS2 expression is likely harmful in kidney tissue and therefore might be considered a potential biomarker of kidney damage. LWM and HMW HAs play controversial roles in fibrosis (Kaul et al. 2022). Interestingly, HAS2 mainly produces HMW-HA, which tends to inhibit fibrosis, although we detected a greater increase in LMW-HA than in HMW-HA in the XLAS model mice, which is consistent with previous studies in which HAS2 overexpression mainly induced LMW-HA accumulation instead of HMW-HA accumulation (Yang et al. 2019; Selbi et al. 2006). This finding could be explained by the active degradation of HMW HA into LMW-HA by hyaluronidase (Kaul et al. 2022) or free radicals (Šoltés et al. 2006; Berdiaki et al. 2023) in renal tissue. Because a major increase in LMW HA over HMW HA is a crucial activator of HA/CD44/TGFβ signaling and fibrosis in XLAS model mice, the degradation of HMW HA into LMW HA could be a key mechanism to increase the LMW/HMW HA ratio and is therefore worthy of further investigation.

The role of CD44 has rarely been investigated in Alport renal disease, although elevated levels of CD44 have been observed across several different kidney diseases, all of which are linked to fibrotic pathology (Eymael et al. 2018; Sun et al. 2024), suggesting a pivotal role of CD44 in renal fibrosis. Here, we propose that CD44 is a driver gene that promotes TGFβ and fibrosis in patients with Alport renal disease. Therefore, targeting CD44 and its pathways may be a promising therapeutic strategy to hinder fibrosis progression and improve the treatment of renal disease. On the basis of previous reports, TGFβ inhibition can ameliorate AS-related renal damage (Suh et al. 2019; Gross et al. 2003). As an upstream activator, CD44 might be a more effective and specific therapeutic target than TGFβ itself for treating AS-related renal disease.

Despite the clear role of the HA‒CD44 interaction in fibrosis (Xia et al. 2021), the underlying mechanism remains unclear. Previously, McKeown-Longo et al. reported that HA binds CD44 and activates the ERK pathway through cooperation with EGFR (McKeown-Longo and Higgins 2021). In another study, HA activated the Notch pathway through CD44 and TLR4 receptors to promote fibrosis in the liver (Yang et al. 2019). As a supplement, our study revealed that HA can activate the highly profibrotic factor TGFβ through its dynamic receptor CD44, highlighting the role of HA/CD44 in activating TGFβ signaling, which is frequently observed to drive fibrosis in AS renal disease. In addition, TGFβ production was activated by CD44 in both kidney and mesenchymal cells, suggesting that CD44-dependent TGFβ activation probably occurs in many types of cells, resulting in widespread fibrosis across whole renal tissues in Alport patients. Moreover, another study revealed that TGFβ signaling can upregulate CD44 (Chang et al. 2017), suggesting positive feedback in response to CD44-dependent TGFβ activation. This evidence supports the mutual activation of signaling by which CD44 and TGFβ together strongly promote fibrosis.

In comparison with a well-established function of TGFβ1 to promote fibrosis across multiple organs, the roles of TGFβ2 and TGFβ3 in fibrosis are far less characterized. TGFβ2 is generally identified as pro-fibrotic. Whereas TGFβ3 is frequently found to inhibit fibrosis (Escasany et al. 2021), despite the fact that TGFβ3 expression levels are increased in fibrous lung and liver (Sun et al. 2021). Controversially, new evidences also showed that TGFβ3 can be pro-fibrotic(Sun et al. 2021; Wilson 2021). Our study observed a concurrent upregulation of all three isoforms and interestingly a relatively higher upregulation of TGFβ2 than TGFβ1/TGFβ3 in both tissues and cells, possibly suggesting that TGFβ2 may critically contribute to XLAS associated renal fibrosis. The independent role of TGFβ2 and TGFβ3 in XLAS associated renal fibrosis is therefore worthy of further investigation.

Our study revealed that fibroblasts are more sensitive to HA treatment than epithelial cells are, which may result from a higher level of CD44 expression in fibroblasts, suggesting that mesenchymal cells, including fibroblasts and myofibroblasts, which generally express high levels of CD44, might be critically involved in transmitting HA/CD44 signaling and thereby promoting TGFβ and fibrosis. This postulation is consistent with a dominant increase of HA and CD44 IHC/IF signals observed in tubulointerstitial instead of glomerulus in our study (Fig. 2). Our findings not only reveal a novel HA/CD44/TGFβ signaling axis that is implicated in Alport renal fibrosis but also suggest a promising strategy to inhibit renal fibrosis by specifically blocking HA/CD44 activation via the inhibition of HA production, CD44 activation or the HA‒CD44 interaction.

However, our study was limited by the use of only collected renal tissues rather than a living model mouse, which was unavailable. In addition, the renal tissues were merely derived from male mice and the sample size was small. The HEK-293 and HEPM cells do not represent any specific type of kidney cells and hereby are only used to exemplify a proof-of-principle. Moreover, the molecular connection between Col4a5 loss and HAS2 overexpression remains to be explored.

Therefore, future investigations are warranted to overcome these limitations. Rescue experiments requiring a certain period of treatment in living mice are needed to confirm the underlying mechanism and to evaluate the potential of HAS2 and CD44 as therapeutic targets. It will also be translationally informative to compare the therapeutic effect of HAS2/HA-CD44 inhibition with the currently used RAAS blockers in both male and female XLAS model mice. Besides, it’s also necessarily critical to determine whether this HA/CD44/TGFβ pathway activated by Col4a5 deficiency is similarly involved in Col4a3 or Col4a4 deficient mice. A key question will be whether HAS2 overexpression is resulted from a Col4a5 deficiency alone or the aberration of the whole α3α4α5 trimer. Single cell sequencing and spatial transcriptomes will help to illustrate a comprehensive atlas of molecular pathway and cell-cell communications which are spatially and temporally evolving. Further in vitro experiments such as isolating specific kidney cells (e.g. tubular cells, fibroblast, podocytes) and modeling an ex vivo ECM to monitor the pathway transduction will give valuable insight into critical molecular interactions that drive pathogenesis, though these experiments are technically challenging.

Taken together, our findings suggest a potential XLAS renal pathology in which COL4A5 deficiency leads to the upregulation of HAS2 and increased LMW HA levels in the ECM. The increased HA in the renal ECM then becomes a messenger to be spread by activated CD44, which is highly expressed in fibroblasts or other types of mesenchymal cells within the renal microenvironment, leading to the activation of TGFβ and thereby promoting fibrosis.

## Conclusion

This study proposes that COL4A5 deficiency may lead to HAS2 overexpression and HA accumulation to activate CD44-TGFβ signaling, thereby promoting fibrosis, encouraging further tests utilizing HAS2 and CD44 as potential therapeutic targets for XLAS patients.

## Electronic supplementary material

Below is the link to the electronic supplementary material.

Supplementary Material 1

## Data Availability

The RNA-sequencing data has not been deposited in a public repository for now due to a concern of privacy, although the authors make sure that these data will be made available upon reasonable inquiries by contacting the correspondence. The other data concerning experiments such as qPCR, western blotting, HA quantification and immunostaining are included in the manuscript.

## Abbreviations

AS: Alport syndrome
CD44: CD44 molecule
COL4A5: Collagen type IV alpha 5 chain
ECM: Extracellular matrix
ESRD: End-stage renal disease
HA: Hyaluronan
HAS: Hyaluronan synthase
HMW-HA: High molecular weight hyaluronan
HRP: Horseradish peroxidase
LMW-HA: Low molecular weight hyaluronan
PFA: Paraformaldehyde
qPCR: Quantative polymerase chain reaction
RAAS: Renin-angiotensin-aldosterone system
SNL: Sambucus nigra lectin
TGFβ: Transforming growth factor beta
WB: Western blotting
XLAS: X-linked alport syndrome
