# Supplementary material for: Increased HA/CD44/TGFβ signaling implicates in renal fibrosis of a Col4a5 mutant Alport mice
Source: Mol Med. 2025 Mar 12;31:96. doi: 10.1186/s10020-025-01146-0 (PMC11905560; doi:10.1186/s10020-025-01146-0)

**Increased HA/CD44/TGFβ signaling implicates in renal fibrosis of a Col4a5 mutant Alport mice**

**Supplementary Information**

**Primer sequences for qPCR**

| Target | sense（5'-3'） | antisense（5'-3'） |
| --- | --- | --- |
| siCD44-#1 | GGAAGAAGAGACCCAAAUCAUUCdTdG | CAGAAUGAUUUGGGUCUCUUCUUCCdAdC |
| siCD44-#2 | GGUUGUUUCUACCAUCAGAdTdT | UCUGAUGGUAGAAACAACCdTdT |
| siCOL4A5-#1 | GGGUCUCAAUGGAAUGAAAGGdTdT | UUUCAUUCCAUUGAGACCCGGdTdT |
| siCOL4A5-#2 | GCAGAUCAGUGAACAGAAAdTdT | UUUCUGUUCACUGAUCUGCdTdT |

**Small interfering RNA sequences**

| **Gene** | **Species** | **Sequences** | |
| --- | --- | --- | --- |
| *COL4A5* | Human | Forward | CAAAAGGTGATCGTGGTTTCCC |
|  |  | Reverse | GTCCAGGTTGTCCATTTGGTC |
| *HAS1* | Human | Forward | GAGCCTCTTCGCGTACCTG |
|  |  | Reverse | CCTCCTGGTAGGCGGAGAT |
| *HAS2* | Human | Forward | TCCTGGATCTCATTCCTCAGC |
|  |  | Reverse | TGCACTGAACACACCCAAAATA |
| *HAS3* | Human | Forward | GCAGTGTATTAGTGGGCCCTT |
|  |  | Reverse | GCACTTGCTGCCTAGGAACTT |
| *CD44* | Human | Forward | CTGCCGCTTTGCAGGTGTA |
|  |  | Reverse | CATTGTGGGCAAGGTGCTATT |
| *TGFB1* | Human | Forward | CAATTCCTGGCGATACCTCAG |
|  |  | Reverse | GCACAACTCCGGTGACATCAA |
| *TGFB2* | Human | Forward | CAGCACACTCGATATGGACCA |
|  |  | Reverse | CCTCGGGCTCAGGATAGTCT |
| *TGFB3* | Human | Forward | ACTTGCACCACCTTGGACTTC |
|  |  | Reverse | GGTCATCACCGTTGGCTCA |
| *GAPDH* | Human | Forward | GGAGCGAGATCCCTCCAAAAT |
|  |  | Reverse | GGCTGTTGTCATACTTCTCATGG |
| *Has1* | Mouse | Forward | GGCGAGCACTCACGATCATC |
|  |  | Reverse | AGGAGTCCATAGCGATCTGAAG |
| *Has2* | Mouse | Forward | GTACGGTGCCTTTTTAGCCTC |
|  |  | Reverse | TAATCGGGGTTTCAAGGGACT |
| *Has3* | Mouse | Forward | CAATCGCCAGGAAGATACCTAC |
|  |  | Reverse | GGAAATTGCTACGCCACACAA |
| *Cd44* | Mouse | Forward | TCGATTTGAATGTAACCTGCCG |
|  |  | Reverse | CAGTCCGGGAGATACTGTAGC |
| *Tgfb1* | Mouse | Forward | CCACCTGCAAGACCATCGAC |
|  |  | Reverse | CTGGCGAGCCTTAGTTTGGAC |
| *Tgfb2* | Mouse | Forward | CTTCGACGTGACAGACGCT |
|  |  | Reverse | GCAGGGGCAGTGTAAACTTATT |
| *Tgfb3* | Mouse | Forward | CCTGGCCCTGCTGAACTTG |
|  |  | Reverse | TTGATGTGGCCGAAGTCCAAC |
| *Gapdh* | Mouse | Forward | AGGTCGGTGTGAACGGATTTG |
|  |  | Reverse | TGTAGACCATGTAGTTGAGGTCA |

**Supplementary Figure S1. Masson's trichrome staining showing widespread fibrosis in 28-week aged XLAS mouse renal tissue in compare with healthy control.**

Masson's trichrome staining was performed in three pairs of HC and XLAS mice numbered as #1, #2 and #3. (A) Representative microscope images showing Masson staining in renal tissues from these corresponding mice. (B) An Orbit image analysis software (https://www.orbit.bio/) was used to quantify fibrosis based on a tissue quantification protocol provided in the manual (https://www.orbit.bio/tissue-quantification/). The average intensity of blue-stained collagen was calculated to indicate fibrosis in 15 randomly selected glomerulus and tubulointerstitial areas. Scale bar=50 μm, data presented as, ±SD, **, *P*<0.01, ***, *P*<0.001


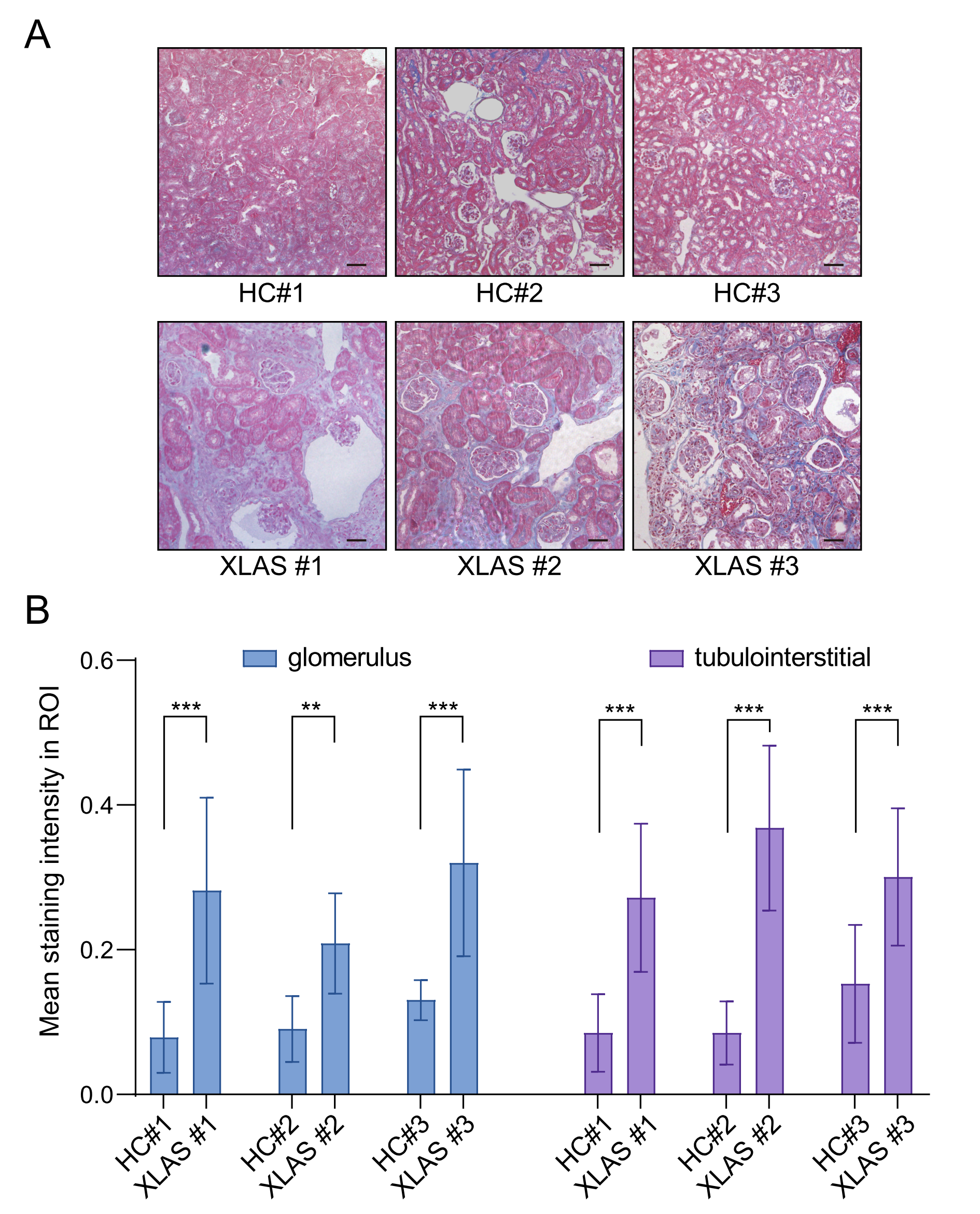


**Supplementary Figure S2. Expression levels of CD44 in HEK-293 and HEPM cells.**

(A). QPCR results comparing the intrinsic CD44 mRNA expression levels between two cell lines. (B). Western blot images contrasting the CD44 protein levels between two cell lines in a single blot


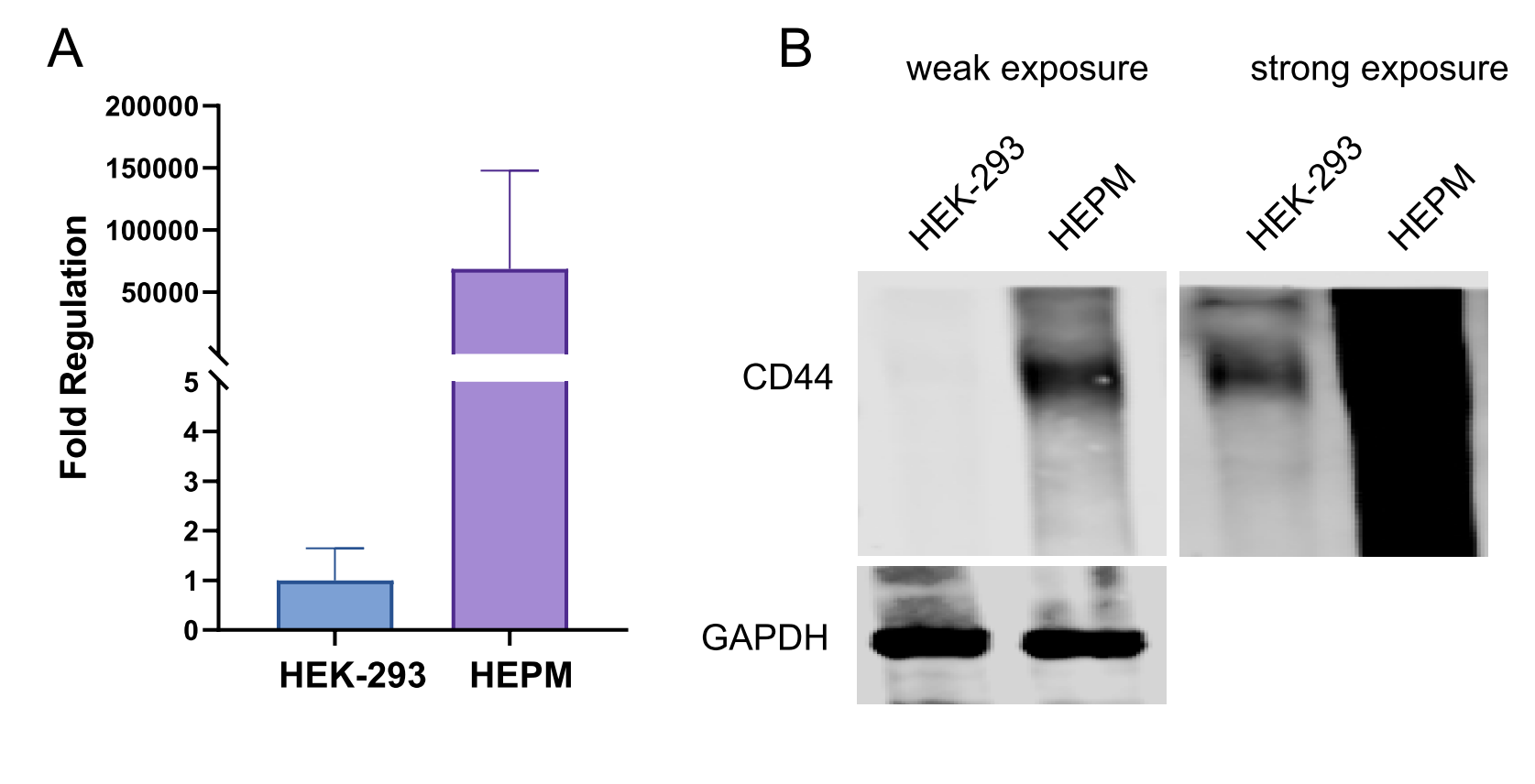


**Supplementary Figure S3. HMW HA did not increase CD44 or TGFβ expression in HEK-293 and HEPM cells.** mRNA levels of CD44, TGFB1, TGFB2, TGFB3 with reference to GAPDH in A. HEK-293 human kidney cells, and B. HEPM human fibroblast cells with or without HMW HA treatment at concentrations of 1 μg/mL and 5 μg/mL.


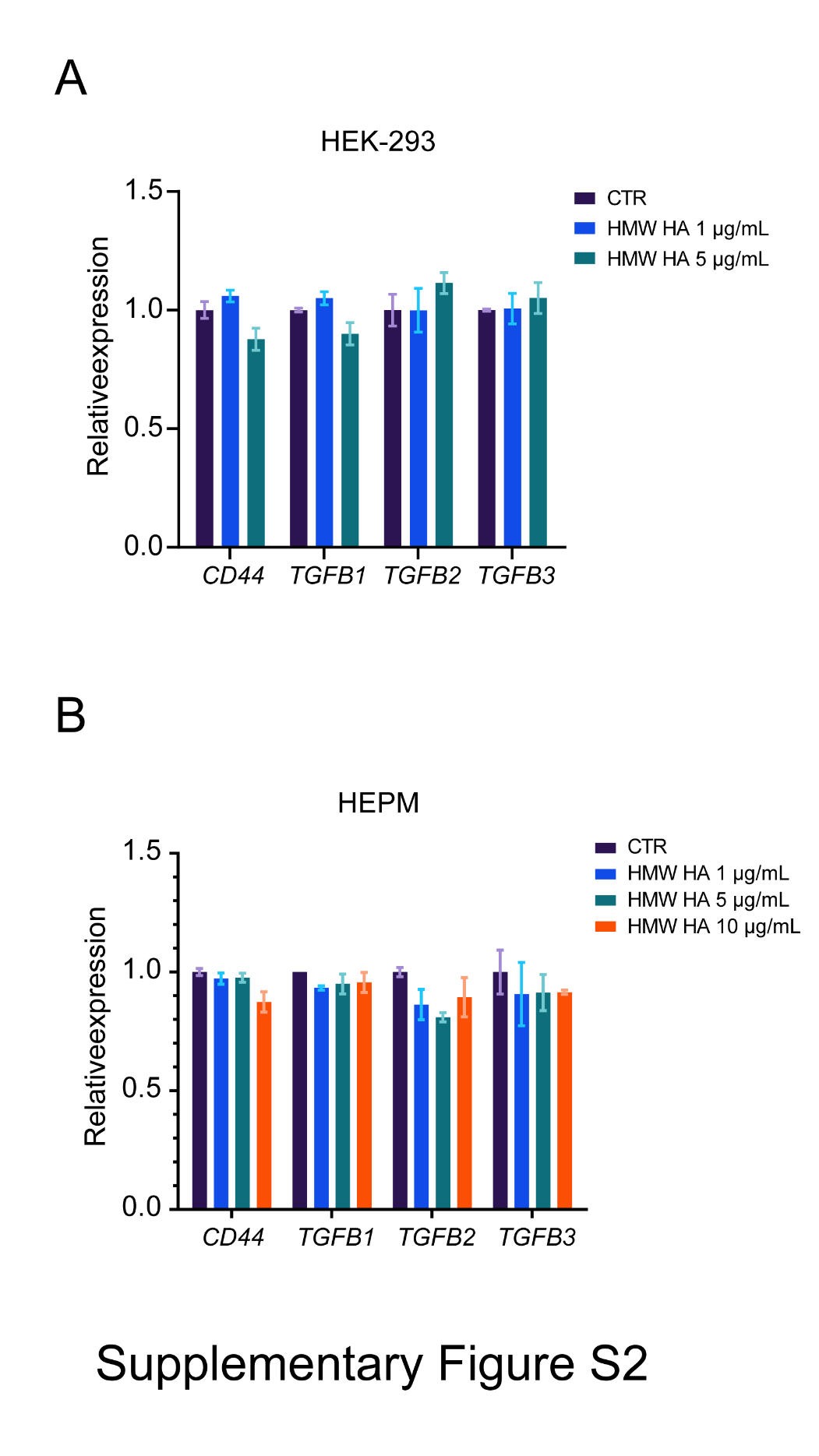

Supplement: Supplementary file 1 — Supplementary Material 1 [file 10020_2025_1146_MOESM1_ESM.docx]
